# Association Analysis of 94 Candidate Genes and Schizophrenia-Related Endophenotypes

**DOI:** 10.1371/journal.pone.0029630

**Published:** 2012-01-13

**Authors:** Tiffany A. Greenwood, Gregory A. Light, Neal R. Swerdlow, Allen D. Radant, David L. Braff

**Affiliations:** 1 Department of Psychiatry, University of California San Diego, La Jolla, California, United States of America; 2 VISN 22 Mental Illness Research, Education and Clinical Centers (MIRECC), Department of Veterans Affairs, San Diego, California, United States of America; 3 Department of Psychiatry and Behavioral Sciences, University of Washington, Seattle, Washington, United States of America; 4 Puget Sound Veterans Administration Health Care System, Seattle, Washington, United States of America; Chiba University Center for Forensic Mental Health, Japan

## Abstract

While it is clear that schizophrenia is highly heritable, the genetic basis of this heritability is complex. Human genetic, brain imaging, and model organism studies have met with only modest gains. A complementary research tactic is to evaluate the genetic substrates of quantitative endophenotypes with demonstrated deficits in schizophrenia patients. We used an Illumina custom 1,536-SNP array to interrogate 94 functionally relevant candidate genes for schizophrenia and evaluate association with both the qualitative diagnosis of schizophrenia and quantitative endophenotypes for schizophrenia. Subjects included 219 schizophrenia patients and normal comparison subjects of European ancestry and 76 schizophrenia patients and normal comparison subjects of African ancestry, all ascertained by the UCSD Schizophrenia Research Program. Six neurophysiological and neurocognitive endophenotype test paradigms were assessed: prepulse inhibition (PPI), P50 suppression, the antisaccade oculomotor task, the Letter-Number Span Test, the California Verbal Learning Test-II, and the Wisconsin Card Sorting Test-64 Card Version. These endophenotype test paradigms yielded six primary endophenotypes with prior evidence of heritability and demonstrated schizophrenia-related impairments, as well as eight secondary measures investigated as candidate endophenotypes. Schizophrenia patients showed significant deficits on ten of the endophenotypic measures, replicating prior studies and facilitating genetic analyses of these phenotypes. A total of 38 genes were found to be associated with at least one endophenotypic measure or schizophrenia with an empirical p-value<0.01. Many of these genes have been shown to interact on a molecular level, and eleven genes displayed evidence for pleiotropy, revealing associations with three or more endophenotypic measures. Among these genes were ERBB4 and NRG1, providing further support for a role of these genes in schizophrenia susceptibility. The observation of extensive pleiotropy for some genes and singular associations for others in our data may suggest both converging and independent genetic (and neural) pathways mediating schizophrenia risk and pathogenesis.

## Introduction

Genetic factors clearly play a substantial role in the etiology of schizophrenia, as evidenced by twin and other family studies that indicate a heritability of up to 80% for this disorder [1]. Despite replicated linkage evidence implicating chromosomes 1q, 5q, 6p, 6q, 8p, 10p, 13q, 15q, and 22q [2] and the identification of several putative susceptibility genes [3], [4], a causative gene or variant for schizophrenia has yet to be definitively identified. One strategy that may aid in identifying the genetic substrates of a complex disorder, like schizophrenia, is to interrogate specific candidate genes thought to be associated with the underlying neurobiology of the disorder or with its associated endophenotypes [5]. To this end, we have constructed a custom SNP array containing 1,536 SNPs in 94 genes that were chosen based on hypotheses regarding biological systems of relevance to schizophrenia, as well as an extensive review of published linkage, association, gene expression, brain imaging, and model organism studies [6]. This custom SNP array provides excellent coverage of many previously suggested and functionally important candidate genes for schizophrenia, including AKT1, CHRNA7, COMT, DAO, DAOA, DISC1, DTNBP1, ERBB4, GRM3, GSK3B, NOS1AP, NRG1, PAFAH1B1, PPP3CC, PRODH, RELN, and RGS4 [3], [4]. Many of the genes represented on the array have also been reported to be involved in brain development and heritable endophenotypes associated with schizophrenia. A similar approach has been used in recent studies of addictive disorders [7] and eating disorders [8], [9].

The aim of the present study was to perform a large-scale candidate gene analysis via this custom SNP array to evaluate the association of six neurophysiological and neurocognitive endophenotypes related to schizophrenia, including prepulse inhibition (PPI) of startle, P50 suppression, the antisaccade task, the California Verbal Learning Task-II (CVLT-II), the Letter-Number Sequencing test (LNS), and the Wisconsin Card Sorting Test-64 Card Version (WCST-64). These endophenotypes were chosen based on demonstrated deficits in schizophrenia patients and prior evidence of reliability, stability, and heritability of the derived measures [5], [10]–[13]. Impaired performance on these endophenotypes has also been demonstrated in clinically unaffected relatives of schizophrenia patients, which provides further evidence that these deficits may reflect part of the heritable risk for the illness [14]–[29]. The endophenotype test paradigms also yielded eight secondary measures, which were evaluated as candidate endophenotypes. We investigate the utility of endophenotypes in facilitating the dissection of the genetic architecture and heritability of schizophrenia [5], [6].

## Methods

### Subject Ascertainment

Subjects were recruited locally through the UCSD Schizophrenia Research Program and included males and females between the ages of 18–65. Schizophrenia outpatients (SZ) were recruited from community board and care facilities and were carefully screened to rule out drug abuse or dependence within the past 6 months and neurologic insults. Diagnoses were confirmed using the Structured Clinical Interview for DSM-IV Axis I Disorders (SCID) [30]. Normal comparison subjects (NCS) answered advertisements and underwent comprehensive clinical interviews via the SCID-Non Patient Edition [31] and SCID-II [32] to rule out other Axis I or II diagnoses (cluster B) and a toxicological screen was performed to rule out current drug abuse. After a detailed description of study participation, written informed consent was obtained for each subject in accordance with protocols 040564 and 071831 as approved by the University of California San Diego Human Research Protections Program.

The case-control sampling strategy provides for a broad range of phenotypic variation in the genetic analyses of these quantitative endophenotypes, since the object of the study was to explore the genetic architecture of quantitative neurophysiological and neurocognitive endophenotypes underlying schizophrenia susceptibility, not necessarily the genetic basis of schizophrenia itself. Additionally, subjects both unaffected and affected with schizophrenia are needed in order to understand how a particular endophenotype contributes to schizophrenia. The current sample includes 322 subjects (203 SZ and 119 NCS). The composition of the sample is approximately 68% subjects of European ancestry, 23% subjects of African ancestry, 4% subjects of Asian ancestry, and 5% of more than one race according to self-reported ethnicity. Note that this sample is completely independent of the previously published Consortium on the Genetics of Schizophrenia (COGS) family-based study [6], [12].

### Phenotypes

Six endophenotype test paradigms were chosen based on their demonstrated deficits in schizophrenia patients and prior evidence of reliability, stability, and heritability of the derived measures [5], [10]–[13]. These endophenotype test paradigms yielded a variety of quantitative measures, including six primary endophenotypes and eight secondary, candidate endophenotypic measures that ranged from largely automatic, neurophysiological measures to highly volitional, neurocognitive measures, as described below [33].

Three neurophysiological endophenotypic test paradigms were assessed. Prepulse inhibition (PPI) of startle was defined as the percent inhibition of the startle reflex caused by a weak prestimulus presented 60 msec prior to a startling stimulus [34]–[36]. We also assessed two secondary measures related to startle: startle magnitude on non-prepulse trials (reactivity) and percent startle habituation from the first to final block of testing. The primary endophenotype of P50 suppression was the ratio of the amplitudes of the P50 event-related potentials generated in response to the conditioning (S1) and test (S2) stimuli presented with a 500 msec interstimulus interval [37]. Secondary measures also considered were the S1–S2 difference, S1 amplitude, and S2 amplitude. The “overlap” antisaccade test of oculomotor inhibition, which assesses the prefrontal-mediated capacity to inhibit a prepotent response, requires subjects to fixate on a central target and respond to a peripheral cue by looking in the opposite direction at the same distance. The primary endophenotype of antisaccade performance was quantified by determining the ratio of correct antisaccades divided by the total number interpretable saccades [38], [39].

Three neurocognitive endophenotypic test paradigms were also assessed. The Letter-Number Span (LNS) is a prototypical task to assess working memory information storage with manipulation. A primary endophenotype was measured as the correct reordering of intermixed numbers and letters (working memory, LNS re-order), and a simple repetition of these letters and numbers in the order dictated was considered a secondary measure (immediate recall, LNS forward) [29], [40]. To assess verbal learning and memory, we used the California Verbal Learning Test, Second Edition (CVLT-II). The primary endophenotype for this test was the total recall score of a list of 16 verbally presented items immediately after presentation summed over 5 trials (immediate recall), and a secondary measure of recall after a 20-minute delay was also considered (delayed recall) [41]. The Wisconsin Card Sorting Test-64 Card Version (WCST-64) [13] was used to assess executive function according to our established procedures [42]. The number of perseverative responses was considered the primary endophenotype with the categories completed as the secondary measure.

### Custom 1,536-SNP Array

We used a candidate gene approach in an attempt to identify genes contributing to the expression of the primary endophenotypes and secondary phenotypic measures. A custom SNP array including 1,536 SNPs within 94 candidate genes was created, as described elsewhere in detail [6], and utilized here. These genes were selected based on complementary information from linkage, association, gene expression, brain imaging, and model organism studies of schizophrenia, as well as knowledge of biological systems particularly relevant to schizophrenia. The resulting array included all of the commonly cited candidate genes for schizophrenia (e.g., COMT, DISC1, DTNBP1, and NRG1), as well as genes from pathways of likely relevance to schizophrenia. Haplotype-tagging SNPs were obtained from the TAMAL website and selected from the HapMap CEU population [43] to efficiently interrogate these genes in our sample of primarily European ancestry. We included 5 kb of flanking sequence on either side of each gene to capture nearby regulatory regions in our tagged regions. Additional SNPs were also included based on prior evidence of association with schizophrenia. The custom array included 1,417 haplotype tagging SNPs for 86 genes, 116 SNPs in 33 genes with reported evidence of association, 29 coding sequence variants in 17 genes (25 nonsynonymous and 4 synonymous), and 18 SNPs located in putative promoter regions or transcription factor binding sites. On average, there was 1 SNP per 10 kb for each gene with variance due to linkage disequilibrium patterns and SNP availability. Minor allele frequencies for these SNPs ranged from 0.01 to 0.50, with an average of 0.23. The complete list of all 1,536 SNPs and 94 candidate genes included on the array and the specific details from our research is available in Table S1 (see References S1 for included references) and elsewhere [6], including rs numbers, chromosomal locations, gene information, designation of SNPs (e.g., as tagging, coding, putatively functional, or associated, including p-values and references), relevant sequence information, and minor allele frequencies for the four HapMap populations. Ingenuity Pathway Analysis (IPA, Ingenuity® Systems) was used to generate a genetic network detailing the interactions between 42 of the 94 genes on the SNP chip, as well as to provide information regarding the clustering of the 94 genes into functional pathways.

### DNA Extraction

Whole blood was drawn from all subjects and placed in anticoagulant (EDTA) tubes for storage at −80°C. The ACCUSPIN System-HISTOPAQUE-1077 (Sigma-Aldrich) was used for the isolation of lymphocytes from the whole blood, and DNA was extracted using PUREGENE DNA Purification Reagents (Gentra). The genomic DNA was quantified using the Quant-iT PicoGreen dsDNA reagent, and purity was assessed by measuring the UV absorbance.

### Genotyping and Cleaning

A total of 203 SZ and 119 NCS were genotyped using 20 µl of genomic DNA at 50 ng/µl plated on 96-well plates with three positive controls per plate, and genotyping was performed by the Biomedical Genomics Laboratory (BIOGEM) at UCSD using an Illumina BeadStation 500 Scanner. Genotype data were cleaned using Illumina's BeadStudio v.3 software for allele calling. Each subject was evaluated across all 1,536 SNPs, and all subjects were found to have acceptable allele call rates, defined as an average call rate >80% and a 50% GenCall Score (median genotype call score) >0.76. Each SNP was then evaluated across all subjects, and 38 SNPs were excluded for having average call rates <90% and cluster separation scores <0.05. Another 95 SNPs were eliminated following a manual examination of all SNPs with call rates >90% but cluster separation scores between 0.05 and 0.25. A total of 133 SNPs were thus removed due to poor allele call rates and/or cluster separation, resulting in a 91.4% SNP assay conversion rate. The final group of 1,403 passing SNPs had a genotype call rate of 99.98%, and accuracy estimated from replicate DNA samples genotyped across the panel indicated a 99.98% reproducibility rate. Further quality control assessments using the PLINK analysis toolset [44] identified two SNPs with Hardy-Weinberg Equilibrium p-values<10^−4^ in the controls and 28 SNPs with minor allele frequencies <0.01 in this sample. Removal of these additional SNPs resulted in 1,373 SNPs for association analysis, the minor allele frequencies of which approximated those observed in the HapMap CEU population. The effective number of independent SNPs for analysis was determined to be 977, after accounting for redundancies in linkage disequilibrium due to the inclusion of putatively functional and/or associated SNPs along with tagging SNPs and gene-spanning SNPs [45].

### Statistical Analyses

Only the ten primary and secondary endophenotypic measures revealing significant (p<0.05) mean differences between the SZ and NCS groups of European ancestry were considered in the association analyses (see Table 1). SZ and NCS groups were combined for these analyses to increase the range of phenotypic variation in each measure. One expects that the phenotypic distributions will overlap between these groups, as some control subjects will show deficits and some SZ subjects will not show deficits for any given measure due to the incomplete correlations of these measures with schizophrenia diagnosis. We have found this to be the case in our sample, and these measures were approximately normally distributed after the removal of outlying values defined as more than three standard deviations from the mean. There were three such outlying values observed for P50 S1 amplitude, one for PPI, and five for startle habituation.

**Table 1:** Descriptive statistics for the primary and secondary endophenotypic measures in the SZ and NCS subjects of European ancestry.

	SZ		NCS		Effect
	N	Mean (SD)	N	Mean (SD)	Size (d)
Age*	126	45.33 (9.02)	92	42.46 (11.11)	−0.29
WRAT-3 Reading Standard Score**	125	94.87 (14.20)	91	107.69 (9.08)	1.06
**Prepulse Inhibition (PPI)** **	**90**	**42.48 (27.22)**	**72**	**55.74 (24.01)**	**0.51**
Startle Magnitude	116	64.24 (56.81)	89	73.39 (50.49)	0.17
**Startle Habituation (Hab)** *	**82**	**48.45 (33.17)**	**71**	**59.00 (28.24)**	**0.34**
P50 Suppression	91	52.37 (31.97)	79	56.66 (27.29)	0.14
P50 S1–S2 Difference	90	1.50 (1.13)	78	1.85 (1.41)	0.28
**P50 S1 Amplitude (P50-S1)** *	**89**	**2.75 (1.34)**	**79**	**3.33 (1.94)**	**0.36**
P50 S2 Amplitude	89	1.23 (0.90)	80	1.42 (1.05)	0.2
**Antisaccade** **	**107**	**0.53 (0.26)**	**89**	**0.80 (0.21)**	**1.14**
**LNS Immediate Recall (LNS-fwd)** **	**125**	**12.28 (3.20)**	**92**	**14.18 (3.06)**	**0.60**
**LNS Working Memory (LNS-reorder)** **	**125**	**7.82 (2.75)**	**92**	**11.33 (2.58)**	**1.31**
**CVLT-II Immediate Recall (CVLT-immed)** **	**125**	**35.88 (11.01)**	**92**	**53.75 (9.75)**	**1.71**
**CVLT-II Delayed Recall (CVLT-delay)** **	**125**	**7.64 (3.38)**	**92**	**11.96 (2.96)**	**1.35**
**WCST-64 Perseverative Responses (WCST-persev)** **	**123**	**21.33 (16.77)**	**92**	**11.00 (9.18)**	**−0.76**
**WCST-64 Categories Completed (WCST-cat)** **	**124**	**2.03 (1.57)**	**92**	**3.43 (1.52)**	**0.90**

Phenotypes with significant differences between the schizophrenia patient (SZ) and normal comparison subject (NCS) groups are indicated in bold.

*p<0.05;

**p<0.001.

Multidimensional scaling (MDS), implemented in PLINK [44], was used to assess the degree of population stratification in this sample and to validate the self-reported subject ethnicities, which are not always reliable. Based on a comparison of the MDS results and the self-reported ethnicities, the largest and most genetically homogenous group included subjects of European ancestry, which formed 68% of the sample and encompassed 219 subjects (127 SZ and 92 NCS). This group of subjects was selected for the primary analyses of the phenotypic measures. We anticipate >80% power to detect a locus explaining 5% of the trait variation at a p-value<0.01 and 10% of the variation at a p-value<1×10^−4^ in this sample. A secondary sample of 76 subjects of African ancestry (62 SZ and 14 NCS) was chosen for follow-up analyses of the most salient findings in the subjects of European ancestry. Subjects of Asian ancestry were very few in number. Those who reported ancestry of more than one race that did not cluster with either the subjects of European or African ancestry in the MDS analysis were also few in number and genetically heterogeneous. These 28 total subjects were thus eliminated from further analysis.

Association analyses between the SNPs and the ten primary and secondary endophenotypic measures were conducted using linear regression methods in PLINK [44], whereas logistic regression methods were employed for the analysis of schizophrenia diagnosis. Age and sex were explored as covariates for all endophenotypes via correlation analyses and incorporated into the association analyses when significant as follows: both age and sex for P50 S1 amplitude, the antisaccade task, LNS re-order, CVLT-II immediate and delayed recall, and WCST-64 perseverative responses; and sex only for WCST-64 categories complete. The first two MDS principal components were also used as covariates in all association analyses of the European and African ancestry subjects separately to correct for any residual population stratification within the two groups.

We did not consider years of education or scores from the Wide Range Achievement Test (WRAT) as potential covariates in these analyses. While education is an important correlate of neurocognitive abilities that may confound genetic association findings, the handling of potential covariates with substantive group differences, such as that demonstrated by education, is not trivial. Schizophrenia patients typically display lower-than-normal levels of education and significantly reduced performances across all neurocognitive measures, as is demonstrated in this sample. Since schizophrenia is a neurodevelopmental disorder characterized by heritable cognitive deficits that are detectable prior to the onset of illness, it is not possible to disentangle the confounding multivariate inter-relationships of schizophrenia status, cognitive abilities, years of formal education, and genetic risk. The use of factors as covariates that are directly impacted by, and may in fact be intrinsic to, schizophrenia would effectively control for case status in this case-control study.

Label-switching permutation procedures were utilized to generate empirical significance levels. Permutations also provide for a more accurate assessment of the association of lower frequency alleles (e.g., minor allele frequencies of 0.01–0.10) with a modest sample size. For each SNP and phenotypic measure, permutations were performed in an adaptive fashion such that the number of permutations performed for a given SNP was relative to the original significance value, with more permutations performed for smaller (more significant) p-values. All association p-values presented are empirical and the result of these adaptive permutations. While all SNPs with minor allele frequencies >0.01 were included in the association analyses, we only present the results for the more common SNPs with frequencies >0.05. The complete results can be found in Table S1, including the SNPs with frequencies <0.05.

## Results

Prior to conducting association analyses, we performed some initial assessments of the primary endophenotypes and secondary endophenotypic measures to validate their informativity in the 219 subjects of European ancestry. Table 1 lists the mean values for all quantitative phenotypic measures in the SZ and NCS groups, as well as the significance of the mean differences. All neurocognitive measures from the LNS (forward and re-order), CVLT-II (immediate and delayed recall), and WCST-64 (perseverative responses and categories completed) revealed robust and highly significant differences (d = 0.60 to d = 1.71, p<0.001) between the SZ and NCS groups. Of the neurophysiological measures, the antisaccade task and PPI revealed highly significant differences (d = 1.14 and d = 0.51, respectively, p<0.001), whereas startle habituation and P50 S1 amplitude revealed more modest but still significant differences (d = 0.36, p<0.05). The remaining neurophysiological measures (i.e., startle magnitude, P50 suppression and difference score, and P50 S2 amplitude) revealed only small differences (d<0.28) between the SZ and NCS groups and were thus excluded from further analyses.

We also assessed the degree of correlation between the endophenotypic measures in the subjects of European ancestry, as shown in Table 2. Age and sex were also assessed as potential covariates, and both were at least moderately associated with most measures (see Table 2). The correlational analyses among endophenotypic measures revealed a pattern of robust (p<0.001) inter-correlations among the majority of neurocognitive measures and the antisaccade task. Other neurophysiological measures, however, revealed fewer and more modest correlations with the neurocognitive measures. No significant correlations were observed among the neurophysiological measures.

**Table 2:** Correlations between the significantly different endophenotypic measures in the SZ and NCS subjects of European ancestry.

	Age	Sex	PPI	Hab	P50-S1	Anti-saccade	LNS-fwd	LNS-reorder	CVLT-immed	CVLT-delay	WCST-persev
PPI	ns	ns									
Hab	ns	ns	ns								
P50-S1	−0.20*	0.17*	ns	ns							
Antisaccade	−0.19*	0.14*	ns	ns	ns						
LNS-fwd	ns	ns	0.18*	ns	0.20*	0.23*					
LNS-reorder	−0.16*	0.24*	0.29**	ns	ns	0.46**	0.56**				
CVLT-immed	−0.25**	0.31*	0.23*	0.19*	0.18*	0.51**	0.36**	0.70**			
CVLT-delay	−0.23*	0.26*	0.18*	0.18*	ns	0.37**	0.28**	0.60**	0.86**		
WCST-persev	0.20*	−0.14*	ns	ns	ns	−0.34**	−0.21*	−0.40**	−0.43**	−0.34**	
WCST-cat	ns	0.16*	ns	ns	ns	0.43**	0.26**	0.47**	0.53**	0.45**	−0.75**

Key: ns = not significant;

*p<0.05;

**p<0.001.

**Figure 1:**
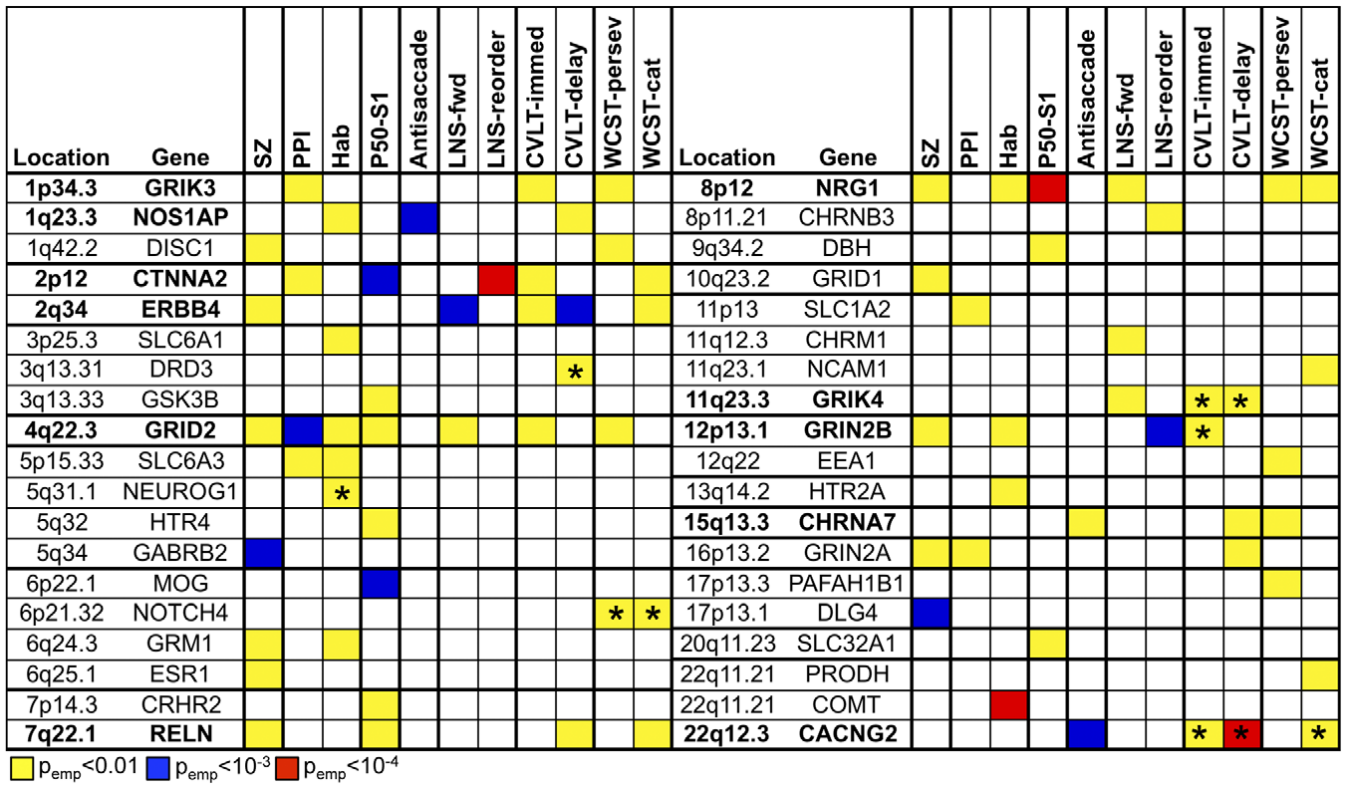
Summary of the most significant associations observed in the European ancestry sample. Empirical p-values are presented for each of the 38 genes with each of the 10 phenotypes and schizophrenia using a minimum empirical p-value of <0.01 as a threshold. Note that not all associations to the same gene across phenotypes reflect associations to the same SNP, although many do. An asterisk (*) indicates that at least one SNP in the gene associated with the specified phenotype has been previously associated with schizophrenia as follows: rs963468 in DRD3 [46], rs2344485 in NEUROG1 [47]; rs520692 in NOTCH4 [48], [49]; rs1954787 in GRIK4 [50]; rs1805247 in GRIN2B [51], [52]; and rs2267341 and rs2283981 in CACNG2 [53]. Genes associated with three or more phenotypes are indicated in bold.

Analysis of the ten endophenotypic measures that significantly differentiated the SZ and NCS groups revealed associations with 34 of the 94 genes collectively, with the qualitative schizophrenia diagnosis revealing associations to four additional genes. Figure 1 provides a summary of the minimum empirical p-values for each gene and endophenotype, and the complete set of results is presented in Table S1. Among these 38 genes, there were four SNPs with empirical p-values<10^−4^, 14 SNPs with empirical p-values<10^−3^, and 98 SNPs with empirical p-values<0.01. The most significant finding in these analyses was for a SNP in CTNNA2 with the LNS re-order measure, which gave an empirical p-value of 3.1×10^−5^. Three other SNPs gave empirical p-values<10^−4^ as follows: NRG1 for P50 S1 (p = 7.2×10^−5^), COMT for startle habituation (p = 8.2×10^−5^), and CACNG2 for CVLT-2 delayed recall (p = 5.3×10^−5^). We also found evidence to support association to two nonsynonymous SNPs: NRG1 Arg38Gln gave an empirical p = 6.2×10^−3^ for WCST-64 perseverative responses, and GRIN2B His1399His gave an empirical p = 6.6×10^−3^ for CVLT-II immediate recall.

The custom SNP chip includes a total of 40 genes that have shown prior allelic or haplotypic associations with schizophrenia or related phenotypes [6]. In our analyses, we found further evidence for association to CACNG2, CHRNA7, COMT, DISC1, DRD3, ERBB4, GABRB2, GRID1, GRIK3, GRIK4, GRIN2B, HTR2A, NCAM1, NEUROG1, NOTCH4, NRG1, PRODH, SLC1A2, and SLC6A3, as detailed in Figure 1, including associations to ten specific SNPs with previous reports of association to schizophrenia [46]–[53]. In contrast to our expectations, we did not find evidence for association to ADRBK2, AKT1, BDNF, DAO, DAOA, DGCR2, DRD2, DRD4, DTNBP1, GAD1, GRIN1, GRM3, GRM4, HTR7, PPP1R1B, PPP3CC, RGS4, SLC18A1, SP4, TAAR6, or ZDHHC8, despite previous reports.

Figure 1 also highlights the associations of genes across the endophenotypic measures. Eleven genes displayed extensive evidence for pleiotropy, revealing associations with three or more phenotypes and often with schizophrenia as well. These genes included GRIK3, NOS1AP, CTNNA2, ERBB4, GRID2, RELN, NRG1, GRIK4, GRIN2B, CHRNA7, and CACNG2. In contrast, other genes were found to be associated with a single endophenotypic measure and/or schizophrenia only. These results may suggest the involvement of multiple pathways in mediating schizophrenia susceptibility.

**Figure 2:**
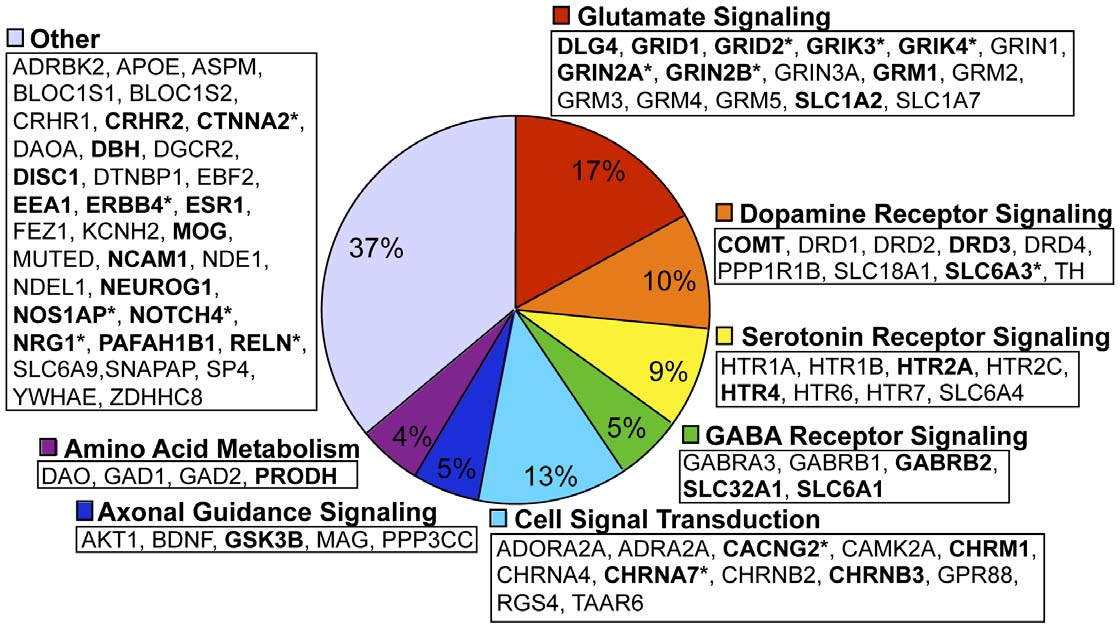
Distribution of the 94 candidate genes in known biological pathways. Associated (empirical p<0.01) genes are indicated in bold, and those associated with more than one phenotype are additionally indicated with an asterisk (*).

**Figure 3:**
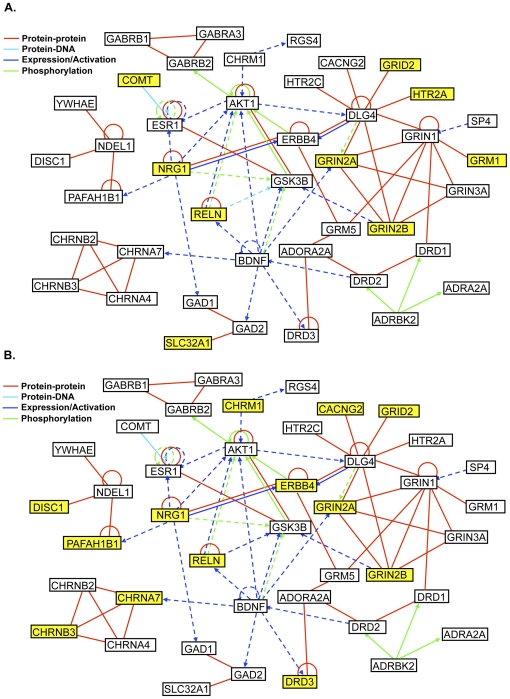
Genetic network detailing the types of interactions between a subset of the 94 candidate genes. Genes associated (empirical p<0.01) with at least one neurophysiological phenotype (PPI, startle habituation, and P50 S1) are highlighted in yellow in (**A**), and genes associated (empirical p<0.01) with the neurocognitive phenotypes (antisaccade, LNS forward, LNS re-order, CVLT-II immediate recall, CVLT-II delayed recall, WCST-64 perseverative responses, or WCST-64 categories) are highlighted in (**B**). Note that antisaccade was grouped with the neurocognitive phenotypes based on its demonstrated correlations with these measures (see Table 2). Genes are represented as nodes, and the biological relationship between two nodes is represented as an edge (line or arrow) supported by at least one reference from the literature, a textbook, or canonical information derived from the human, mouse, and rat orthologs of the gene that are stored in the Ingenuity Pathways Knowledge Base. Solid and dashed lines/arrows indicate direct and indirect interactions, respectively.

As expected, the 94 candidate genes on the chip cluster into multiple pathways thought to be of relevance to schizophrenia, which is a highly heterogeneous disorder. These included cell signal transduction, axonal guidance, amino acid metabolism, and dopamine, GABA, glutamate, and serotonin receptor signaling. The 38 genes significantly associated with at least one endophenotypic measure or schizophrenia itself are distributed amongst these pathways, as shown in Figure 2. There is a notable cluster of associated genes in the glutamate signaling pathway where 9 of the 16 genes revealed associations to at least one phenotype, and five genes (GRID2, GRIK3, GRIK4, GRIN2A, and GRIN2B) were associated with more than one endophenotypic measure. We also explored the underlying molecular interactions between a subset of the 94 genes on the custom SNP chip using Ingenuity Pathway Analysis, as shown in Figure 3. Networks detailing the interactions between genes found to be associated with at least one neurophysiological or neurocognitive measure are highlighted separately and reveal overlapping yet distinct patterns of gene involvement between the neurophysiological and neurocognitive phenotypic domains.

**Figure 4:**
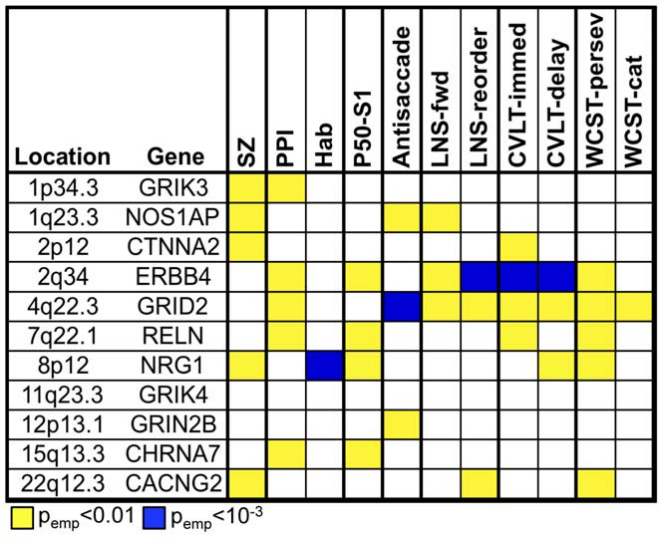
Summary of the most significant associations observed in the African ancestry sample. Empirical p-values are presented for each of the 11 genes exhibiting pleiotropy in the analyses of the European ancestry sample with each of the 10 phenotypes and schizophrenia using a minimum empirical p-value of <0.01 as a threshold. Note that not all associations to the same gene across phenotypes reflect associations to the same SNP, although many do.

We attempted to replicate and extend these findings in the subjects of African ancestry collected as part of this sample. We included for analysis only those 11 genes that revealed extensive evidence for pleiotropy in the European ancestry sample as discussed above. As shown in Figure 4, we found further evidence to support association to all but GRIK4 (see Table S1 for a complete description of the results). Several of the genes (GRIK3, NOS1AP, CTNNA2, ERBB4, GRID2, RELN, NRG1) revealed a similar pattern of associations across the endophenotypic measures to that observed in the European ancestry sample. While most of the genes were associated with more than one endophenotypic measure, four genes (ERBB4, GRID2, RELN, and NRG1) once again displayed extensive pleiotropy with associations to four or more endophenotypic measures.

## Discussion

In this study, we assessed six neurophysiological and neurocognitive primary endophenotypes with prior evidence of heritability and demonstrated schizophrenia-related impairments, as well as eight secondary endophenotypic measures derived from the endophenotype test paradigms. Ten of the endophenotypic measures successfully differentiated between schizophrenia patients and controls. Analysis of these endophenotypic measures revealed an expected pattern of robust correlations (p<0.001) among the neurocognitive phenotypes, consistent with many studies demonstrating a generalized and inter-dependent pattern of neurocognitive deficits in schizophrenia (e.g. [54]–[60]). The antisaccade task was also significantly correlated with the neurocognitive phenotypes, whereas other neurophysiological measures revealed expectedly fewer and more moderate correlations with the neurocognitive measures [33], [37], [61]. However, no significant correlations were observed among the neurophysiological endophenotypes, suggesting that these measures represent independent neurobiological processes with distinct neural and genetic substrates.

Association analyses of the ten endophenotypic measures differing between schizophrenia patients and controls identified both singular genetic associations, as well as genes exhibiting pleiotropic effects across several endophenotypic domains. Specifically, we observed associations between the ten endophenotypic measures and 36 genes thought to be of biological relevance to schizophrenia (see Figure 1). Since the genes on the chip were chosen based on neurobiological relevance and prior association with schizophrenia, those revealing associations with multiple endophenotypic measures may be of particular interest. Indeed, ERBB4, GRID2, RELN, and NRG1 in particular revealed extensive pleiotropy across the endophenotypic measures in both the European and African ancestry samples, offering a compelling picture of the global importance of these genes in the neuropathology of schizophrenia and its associated heritable deficits. We also observed a notable cluster of associated genes in the glutamate signaling pathway, which is consistent with other reports that have shown genes in cellular signaling and neurodevelopmental processes, including the neuregulin and glutamate pathways, to be disproportionately disrupted in schizophrenia [6], [62]. Overall, the observation of extensive pleiotropy for some genes and singular associations for others in our data may suggest the presence of both overlapping and distinct pathways mediating schizophrenia pathogenesis.

This pattern of results is similar to that seen in the recently published analyses of an independent family-based sample from the Consortium on the Genetics of Schizophrenia (COGS) [6]. While both the COGS study and that presented here have utilized the same 1,536 custom SNP array for the assessment of genetic associations with neurophysiological and neurocognitive endophenotypes for schizophrenia, the particular endophenotypes assessed, the ascertainment schemes employed, and the computational methods used for analysis are quite distinct between the two studies. The COGS ascertained families through probands with schizophrenia who had at least one unaffected sibling and both parents available for testing and used variance component methods to evaluate genetic associations of the quantitative endophenotypes in their family-based sample. For the current study, we recruited all available schizophrenia patients regardless of family availability, along with healthy controls, and used linear regression methods to evaluate genetic associations of the quantitative endophenotypes in our sample of unrelated subjects. The COGS study also used a novel bootstrap method to correct for multiple comparisons, since simple permutation schemes, such as that used here, cannot accommodate family-based samples with quantitative traits and covariates. The combination of the aforementioned differences makes a point-by-point comparison of the results of these two studies difficult. Nevertheless, if we compare the overall results of the current study with those from the COGS study, we find that nine genes feature very prominently in both samples: GRIK3, NOS1AP, CTNNA2, ERBB4, GRID2, RELN, NRG1, GRIK4, and GRIN2B. We also find a total of 28 genes that are associated with at least one endophenotype in both samples, many of which cluster in the glutamate pathway. Collectively, these results support a strong role for genes involved in glutamate signaling in mediating schizophrenia susceptibility and/or endophenotype deficits.

With the analysis of 94 candidate genes and ten endophenotypic measures, the issue of multiple comparisons must be considered. However, correction for multiple testing is not trivial in this case, since many of the ten endophenotypes are significantly inter-correlated and are therefore cannot be considered independent (see Table 2). This fact makes a correction for multiple phenotypic comparisons challenging. Further complicating the issue, 40 of the genes on the chip (42%) were selected based on a priori evidence of association to schizophrenia in the literature, so the analyses of these genes could be considered “modified replication studies.” Since a p-value of 0.05 is often considered an adequate threshold for replication, applying a Bonferroni correction based on the total number of SNPs analyzed (977) and requiring a p-value of 5×10^−5^ for significance, is clearly not appropriate and is overly conservative in this type of situation. We have thus utilized permutation procedures to provide for a more accurate assessment of association between each locus and endophenotypic measure. We have also analyzed a subset of the genes identified in the subjects of European ancestry in an independent, albeit small, sample of African ancestry to replicate and extend the initial results in a genetically distinct population.

A possible limitation of this study is the assessment of lower frequency SNPs through linear regression in a sample of modest size. Permutation procedures were used to provide a more accurate assessment of the observed associations, which is particularly critical for lower frequency SNPs. However, one might argue that a higher minor allele threshold for inclusion should be used. There are 85 SNPs with minor allele frequencies of 1–5%, three of which are nonsynonymous coding variants of potentially high interest. While we have assessed all SNPs meeting our allele frequency threshold of 1%, we highlight only the results for SNPs with frequencies of at least 5%, providing the complete results in Table S1.

Studies of disorders as heterogeneous as schizophrenia are replete with failures to replicate findings. The selected endophenotypes themselves also present several challenges. For example, molecular, animal model, and human genetic studies of P50 suppression deficits in schizophrenia present an elegant and logical picture. However, in this study, P50 suppression measured both by ratio and difference score methods failed to reveal significant differences between the SZ and NCS groups. In contrast, schizophrenia patients showed a significantly diminished “S1” response to the first of the two-click paired stimuli. Moreover, important methodological differences might also account for our failure to detect significant P50 gating deficits in this sample as well as across other laboratories [63]. Additionally, antipsychotic medications may affect these results, although they tend to “normalize” endophenotypic scores (e.g. [35], [64]), thereby reducing, rather than increasing, the probability of association. Genetic analyses of schizophrenia are also plagued by nonreplication (e.g. [65]), despite the striking heritability of the disorder [1]. Here, too, we found no evidence for association to some prominent schizophrenia candidate genes, such as DAO, DAOA, DTNBP1, PPP3CC, and RGS4 [3], [4]. These inconsistencies are understandable in the context of ascertainment biases, population stratification, and cohort variance due to gender, smoking, treatment, age of onset, and a plethora of other factors. The present sample size also does not provide the statistical power to detect more modest gene-phenotype associations and definitively identify non-associations. Additionally, the degree of allelic, locus, and phenotypic heterogeneity in schizophrenia patients now appears to be far more extensive than previously appreciated, which, combined with emerging evidence for epigenetic effects and many individual-specific rare variants, may have important implications for gene discovery [66], [67].

Overall, these data reflect and extend our knowledge of the genetic basis of neurophysiological and neurocognitive endophenotypes related to schizophrenia and of schizophrenia itself. Each study into this research domain should be viewed as one building block in constructing a comprehensive picture of the genetic basis of schizophrenia. Further analyses of the genes associated with each of these endophenotypes may provide additional information regarding the underlying genetic pathways involved in schizophrenia susceptibility and endophenotype deficits. By further refining the observed associations with each endophenotype, identifying the underlying causal genetic variants, and elaborating their molecular interactions, the field will be better positioned to understand the underlying genetics and neuropathology of this common, polygenic disorder and hopefully to facilitate early identification and individualized treatment strategies for schizophrenia patients.

## Supporting Information

Table S1 Summary of all 1,536 SNPs present on the custom SNP array and association results for the European ancestry and African ancestry samples. Key: OR = odds ratio; Beta = beta statistic for quantitative association; P = empirical p value; Eur = results for the European ancestry sample; Afr = results for the African ancestry sample; MAF = minor allele frequency; H-W = Hardy-Weinberg p value; htSNP = haplotype tagging SNP (T = TAGGER SNP selection method; G = Gabriel SNP selection method); Coding = nonsynonymous or synonymous coding sequence variant; Prom = promoter variant; TFBS = transcription factor binding site variant; Association = SNP with prior evidence of association (see Supporting References); CEU = CEPH/Caucasian; YRI = Yoruban/African; JPT = Japanese; HCB = Chinese. *Y = yes.
(XLS)

References S1 Complete listing of all references included in Table S1.
(DOC)
