# Supplementary material for: Association Analysis of 94 Candidate Genes and Schizophrenia-Related Endophenotypes
Source: PLoS One. 2012 Jan 13;7(1):e29630. doi: 10.1371/journal.pone.0029630 (PMC3258248; doi:10.1371/journal.pone.0029630)
Supplement: References S1 — Complete listing of all references included in Table S1. (DOC) [file pone.0029630.s002.doc]

**REFERENCES S1**

1. Shibata H, Aramaki T, Sakai M, Ninomiya H, Tashiro N, et al. (2006) Association study of polymorphisms in the GluR7, KA1 and KA2 kainate receptor genes (GRIK3, GRIK4, GRIK5) with schizophrenia. Psychiatry Res 141: 39-51.
2. Chowdari KV, Mirnics K, Semwal P, Wood J, Lawrence E, et al. (2002) Association and linkage analyses of RGS4 polymorphisms in schizophrenia. Hum Mol Genet 11: 1373-1380.
3. Chen X, Dunham C, Kendler S, Wang X, O'Neill FA, et al. (2004) Regulator of G-protein signaling 4 (RGS4) gene is associated with schizophrenia in Irish high density families. Am J Med Genet B Neuropsychiatr Genet 129: 23-26.
4. Talkowski ME, Seltman H, Bassett AS, Brzustowicz LM, Chen X, et al. (2006) Evaluation of a susceptibility gene for schizophrenia: genotype based meta-analysis of RGS4 polymorphisms from thirteen independent samples. Biol Psychiatry 60: 152-162.
5. Morris DW, Rodgers A, McGhee KA, Schwaiger S, Scully P, et al. (2004) Confirming RGS4 as a susceptibility gene for schizophrenia. Am J Med Genet B Neuropsychiatr Genet 125: 50-53.
6. Fallin MD, Lasseter VK, Avramopoulos D, Nicodemus KK, Wolyniec PS, et al. (2005) Bipolar I disorder and schizophrenia: a 440-single-nucleotide polymorphism screen of 64 candidate genes among Ashkenazi Jewish case-parent trios. Am J Hum Genet 77: 918-936.
7. Zhang F, Sarginson J, Crombie C, Walker N, St Clair D, et al. (2006) Genetic association between schizophrenia and the DISC1 gene in the Scottish population. Am J Med Genet B Neuropsychiatr Genet 141: 155-159.
8. Hennah W, Varilo T, Kestila M, Paunio T, Arajarvi R, Haukka J, et al. (2003): Haplotype transmission analysis provides evidence of association for DISC1 to schizophrenia and suggests sex-dependent effects. *Hum Mol Genet*. 12:3151-3159.
9. Liu YL, Fann CS, Liu CM, Chen WJ, Wu JY, et al. (2006) A single nucleotide polymorphism fine mapping study of chromosome 1q42.1 reveals the vulnerability genes for schizophrenia, GNPAT and DISC1: Association with impairment of sustained attention. Biol Psychiatry 60: 554-562.
10. Chen QY, Chen Q, Feng GY, Lindpaintner K, Wang LJ, et al. (2007) Case-control association study of Disrupted-in-Schizophrenia-1 (DISC1) gene and schizophrenia in the Chinese population. J Psychiatr Res 41: 428-434.
11. Qu M, Tang F, Yue W, Ruan Y, Lu T, et al. (2007) Positive association of the Disrupted-in-Schizophrenia-1 gene (DISC1) with schizophrenia in the Chinese Han population. Am J Med Genet B Neuropsychiatr Genet 144: 266-270.
12. Callicott JH, Straub RE, Pezawas L, Egan MF, Mattay VS, et al. (2005) Variation in DISC1 affects hippocampal structure and function and increases risk for schizophrenia. Proc Natl Acad Sci U S A 102: 8627-8632.
13. DeRosse P, Hodgkinson CA, Lencz T, Burdick KE, Kane JM, et al. (2007) Disrupted in schizophrenia 1 genotype and positive symptoms in schizophrenia. Biol Psychiatry 61: 1208-1210.
14. Addington AM, Gornick M, Duckworth J, Sporn A, Gogtay N, et al. (2005) GAD1 (2q31.1), which encodes glutamic acid decarboxylase (GAD67), is associated with childhood-onset schizophrenia and cortical gray matter volume loss. Mol Psychiatry 10: 581-588.
15. Silberberg G, Darvasi A, Pinkas-Kramarski R, Navon R (2006) The involvement of ErbB4 with schizophrenia: association and expression studies. Am J Med Genet B Neuropsychiatr Genet 141: 142-148.
16. Talkowski ME, Mansour H, Chowdari KV, Wood J, Butler A, et al. (2006) Novel, replicated associations between dopamine D3 receptor gene polymorphisms and schizophrenia in two independent samples. Biol Psychiatry 60: 570-577.
17. Dominguez E, Loza MI, Padin F, Gesteira A, Paz E, et al. (2007) Extensive linkage disequilibrium mapping at HTR2A and DRD3 for schizophrenia susceptibility genes in the Galician population. Schizophr Res 90: 123-129.
18. Greenwood TA, Schork NJ, Eskin E, Kelsoe JR (2006) Identification of additional variants within the human dopamine transporter gene provides further evidence for an association with bipolar disorder in two independent samples. Mol Psychiatry 11: 125-133.
19. Fanous AH, Chen X, Wang X, Amdur RL, O'Neill FA, et al. (2007) Association between the 5q31.1 gene neurogenin1 and schizophrenia. Am J Med Genet B Neuropsychiatr Genet 144: 207-214.
20. Lo WS, Lau CF, Xuan Z, Chan CF, Feng GY, et al. (2004) Association of SNPs and haplotypes in GABAA receptor beta2 gene with schizophrenia. Mol Psychiatry 9: 603-608.
21. Lo WS, Harano M, Gawlik M, Yu Z, Chen J, et al. (2007) GABRB2 association with schizophrenia: commonalities and differences between ethnic groups and clinical subtypes. Biol Psychiatry 61: 653-660.
22. Yu Z, Chen J, Shi H, Stoeber G, Tsang SY, et al. (2006) Analysis of GABRB2 association with schizophrenia in German population with DNA sequencing and one-label extension method for SNP genotyping. Clin Biochem 39: 210-218.
23. Straub RE, Jiang Y, MacLean CJ, Ma Y, Webb BT, et al. (2002) Genetic variation in the 6p22.3 gene DTNBP1, the human ortholog of the mouse dysbindin gene, is associated with schizophrenia. Am J Hum Genet 71: 337-348.
24. Li T, Zhang F, Liu X, Sun X, Sham PC, et al. (2005) Identifying potential risk haplotypes for schizophrenia at the DTNBP1 locus in Han Chinese and Scottish populations. Mol Psychiatry 10: 1037-1044.
25. Schwab SG, Knapp M, Mondabon S, Hallmayer J, Borrmann-Hassenbach M, et al. (2003) Support for association of schizophrenia with genetic variation in the 6p22.3 gene, dysbindin, in sib-pair families with linkage and in an additional sample of triad families. Am J Hum Genet 72: 185-190.
26. van den Oord EJ, Sullivan PF, Jiang Y, Walsh D, O'Neill FA, et al. (2003) Identification of a high-risk haplotype for the dystrobrevin binding protein 1 (DTNBP1) gene in the Irish study of high-density schizophrenia families. Mol Psychiatry 8: 499-510.
27. Stefanis NC, Trikalinos TA, Avramopoulos D, Smyrnis N, Evdokimidis I, et al. (2007) Impact of schizophrenia candidate genes on schizotypy and cognitive endophenotypes at the population level. Biol Psychiatry 62: 784-792.
28. Funke B, Finn CT, Plocik AM, Lake S, DeRosse P, et al. (2004) Association of the DTNBP1 locus with schizophrenia in a U.S. population. Am J Hum Genet 75: 891-898.
29. Wang Z, Wei J, Zhang X, Guo Y, Xu Q, et al. (2006) A review and re-evaluation of an association between the NOTCH4 locus and schizophrenia. Am J Med Genet B Neuropsychiatr Genet 141: 902-906.
30. Zhang X, Wei J, Yu YQ, Liu SZ, Shi JP, et al. (2004) Is NOTCH4 associated with schizophrenia? Psychiatr Genet 14: 43-46.
31. Duan J, Martinez M, Sanders AR, Hou C, Saitou N, et al. (2004) Polymorphisms in the trace amine receptor 4 (TRAR4) gene on chromosome 6q23.2 are associated with susceptibility to schizophrenia. Am J Hum Genet 75: 624-638.
32. Pae CU, Yu HS, Amann D, Kim JJ, Lee CU, et al. (2008) Association of the trace amine associated receptor 6 (TAAR6) gene with schizophrenia and bipolar disorder in a Korean case control sample. J Psychiatr Res 42: 35-40.
33. Zhou X, Tang W, Greenwood TA, Guo S, He L, et al. (2009) Transcription factor SP4 is a susceptibility gene for bipolar disorder. PLoS One 4: e5196.
34. Egan MF, Straub RE, Goldberg TE, Yakub I, Callicott JH, et al. (2004) Variation in GRM3 affects cognition, prefrontal glutamate, and risk for schizophrenia. Proc Natl Acad Sci U S A 101: 12604-12609.
35. Chen Q, He G, Wu S, Xu Y, Feng G, et al. (2005) A case-control study of the relationship between the metabotropic glutamate receptor 3 gene and schizophrenia in the Chinese population. Schizophr Res 73: 21-26.
36. Fujii Y, Shibata H, Kikuta R, Makino C, Tani A, et al. (2003) Positive associations of polymorphisms in the metabotropic glutamate receptor type 3 gene (GRM3) with schizophrenia. Psychiatr Genet 13: 71-76.
37. Richards M, Iijima Y, Kondo H, Shizuno T, Hori H, et al. (2006) Association study of the vesicular monoamine transporter 1 (VMAT1) gene with schizophrenia in a Japanese population. Behav Brain Funct 2: 39.
38. Zhao X, Li H, Shi Y, Tang R, Chen W, et al. (2006) Significant association between the genetic variations in the 5' end of the N-methyl-D-aspartate receptor subunit gene GRIN1 and schizophrenia. Biol Psychiatry 59: 747-753.
39. Ikeda M, Iwata N, Kitajima T, Suzuki T, Yamanouchi Y, et al. (2006) Positive association of the serotonin 5-HT7 receptor gene with schizophrenia in a Japanese population. Neuropsychopharmacology 31: 866-871.
40. Neves-Pereira M, Cheung JK, Pasdar A, Zhang F, Breen G, et al. (2005) BDNF gene is a risk factor for schizophrenia in a Scottish population. Mol Psychiatry 10: 208-212.
41. Sullivan PF, Keefe RS, Lange LA, Lange EM, Stroup TS, et al. (2007) NCAM1 and neurocognition in schizophrenia. Biol Psychiatry 61: 902-910.
42. Atz ME, Rollins B, Vawter MP (2007) NCAM1 association study of bipolar disorder and schizophrenia: polymorphisms and alternatively spliced isoforms lead to similarities and differences. Psychiatr Genet 17: 55-67.
43. Pickard BS, Malloy MP, Christoforou A, Thomson PA, Evans KL, et al. (2006) Cytogenetic and genetic evidence supports a role for the kainate-type glutamate receptor gene, GRIK4, in schizophrenia and bipolar disorder. Mol Psychiatry 11: 847-857.
44. Ohtsuki T, Sakurai K, Dou H, Toru M, Yamakawa-Kobayashi K, et al. (2001) Mutation analysis of the NMDAR2B (GRIN2B) gene in schizophrenia. Mol Psychiatry 6: 211-216.
45. Di Maria E, Gulli R, Begni S, De Luca A, Bignotti S, et al. (2004) Variations in the NMDA receptor subunit 2B gene (GRIN2B) and schizophrenia: a case-control study. Am J Med Genet B Neuropsychiatr Genet 128: 27-29.
46. Li D, He L (2007) Association study between the NMDA receptor 2B subunit gene (GRIN2B) and schizophrenia: a HuGE review and meta-analysis. Genet Med 9: 4-8.
47. Liu X, He G, Wang X, Chen Q, Qian X, et al. (2004) Association of DAAO with schizophrenia in the Chinese population. Neurosci Lett 369: 228-233.
48. Schumacher J, Jamra RA, Freudenberg J, Becker T, Ohlraun S, et al. (2004) Examination of G72 and D-amino-acid oxidase as genetic risk factors for schizophrenia and bipolar affective disorder. Mol Psychiatry 9: 203-207.
49. Baritaki S, Rizos E, Zafiropoulos A, Soufla G, Katsafouros K, et al. (2004) Association between schizophrenia and DRD3 or HTR2 receptor gene variants. Eur J Hum Genet 12: 535-541.
50. Golimbet VE, Lavrushina OM, Kaleda VG, Abramova LI, Lezheiko TV (2007) Supportive evidence for the association between the T102C 5-HTR2A gene polymorphism and schizophrenia: a large-scale case-control and family-based study. Eur Psychiatry 22: 167-170.
51. Hattori E, Liu C, Badner JA, Bonner TI, Christian SL, et al. (2003) Polymorphisms at the G72/G30 gene locus, on 13q33, are associated with bipolar disorder in two independent pedigree series. Am J Hum Genet 72: 1131-1140.
52. Chumakov I, Blumenfeld M, Guerassimenko O, Cavarec L, Palicio M, et al. (2002) Genetic and physiological data implicating the new human gene G72 and the gene for D-amino acid oxidase in schizophrenia. Proc Natl Acad Sci U S A 99: 13675-13680.
53. Addington AM, Gornick M, Sporn AL, Gogtay N, Greenstein D, et al. (2004) Polymorphisms in the 13q33.2 gene G72/G30 are associated with childhood-onset schizophrenia and psychosis not otherwise specified. Biol Psychiatry 55: 976-980.
54. Hall D, Gogos JA, Karayiorgou M (2004) The contribution of three strong candidate schizophrenia susceptibility genes in demographically distinct populations. Genes Brain Behav 3: 240-248.
55. Yue W, Kang G, Zhang Y, Qu M, Tang F, et al. (2007) Association of DAOA polymorphisms with schizophrenia and clinical symptoms or therapeutic effects. Neurosci Lett 416: 96-100.
56. Ma J, Qin W, Wang XY, Guo TW, Bian L, et al. (2006) Further evidence for the association between G72/G30 genes and schizophrenia in two ethnically distinct populations. Mol Psychiatry 11: 479-487.
57. Korostishevsky M, Kremer I, Kaganovich M, Cholostoy A, Murad I, et al. (2006) Transmission disequilibrium and haplotype analyses of the G72/G30 locus: suggestive linkage to schizophrenia in Palestinian Arabs living in the North of Israel. Am J Med Genet B Neuropsychiatr Genet 141: 91-95.
58. Ikeda M, Iwata N, Suzuki T, Kitajima T, Yamanouchi Y, et al. (2004) Association of AKT1 with schizophrenia confirmed in a Japanese population. Biol Psychiatry 56: 698-700.
59. Bajestan SN, Sabouri AH, Nakamura M, Takashima H, Keikhaee MR, et al. (2006) Association of AKT1 haplotype with the risk of schizophrenia in Iranian population. Am J Med Genet B Neuropsychiatr Genet 141: 383-386.
60. Schwab SG, Hoefgen B, Hanses C, Hassenbach MB, Albus M, et al. (2005) Further evidence for association of variants in the AKT1 gene with schizophrenia in a sample of European sib-pair families. Biol Psychiatry 58: 446-450.
61. Stephens SH, Logel J, Barton A, Franks A, Schultz J, et al. (2009) Association of the 5'-upstream regulatory region of the alpha7 nicotinic acetylcholine receptor subunit gene (CHRNA7) with schizophrenia. Schizophr Res 109: 102-112.
62. Meyer-Lindenberg A, Straub RE, Lipska BK, Verchinski BA, Goldberg T, et al. (2007) Genetic evidence implicating DARPP-32 in human frontostriatal structure, function, and cognition. J Clin Invest 117: 672-682.
63. Shifman S, Levit A, Chen ML, Chen CH, Bronstein M, et al. (2006) A complete genetic association scan of the 22q11 deletion region and functional evidence reveal an association between DGCR2 and schizophrenia. Hum Genet 120: 160-170.
64. Funke B, Malhotra AK, Finn CT, Plocik AM, Lake SL, et al. (2005) COMT genetic variation confers risk for psychotic and affective disorders: a case control study. Behav Brain Funct 1: 19.
65. Chen X, Wang X, O'Neill AF, Walsh D, Kendler KS (2004) Variants in the catechol-o-methyltransferase (COMT) gene are associated with schizophrenia in Irish high-density families. Mol Psychiatry 9: 962-967.
66. Meyer-Lindenberg A, Nichols T, Callicott JH, Ding J, Kolachana B, et al. (2006) Impact of complex genetic variation in COMT on human brain function. Mol Psychiatry 11: 867-877, 797.
67. Schurhoff F, Szoke A, Chevalier F, Roy I, Meary A, et al. (2007) Schizotypal dimensions: an intermediate phenotype associated with the COMT high activity allele. Am J Med Genet B Neuropsychiatr Genet 144: 64-68.
68. Mukai J, Liu H, Burt RA, Swor DE, Lai WS, et al. (2004) Evidence that the gene encoding ZDHHC8 contributes to the risk of schizophrenia. Nat Genet 36: 725-731.
69. Chen WY, Shi YY, Zheng YL, Zhao XZ, Zhang GJ, et al. (2004) Case-control study and transmission disequilibrium test provide consistent evidence for association between schizophrenia and genetic variation in the 22q11 gene ZDHHC8. Hum Mol Genet 13: 2991-2995.
70. Barrett TB, Emberton JE, Nievergelt CM, Liang SG, Hauger RL, et al. (2007) Further evidence for association of GRK3 to bipolar disorder suggests a second disease mutation. Psychiatr Genet 17: 315-322.
71. Liang SG, Shekhtman T, Gaucher MA, Barrett TB, Schork NJ, et al. (2005) High density SNP association study of 22q13 identifies CACGN2 as a susceptibility locus for bipolar disorder in two independent samples. Am J Med Genet (Neuropsychiatric Genet) 138B: 27.
